# HPV 16 E6/E7 up-regulate the expression of both HIF-1α and GLUT1 by inhibition of RRAD and activation of NF-κB in lung cancer cells

**DOI:** 10.7150/jca.37070

**Published:** 2019-11-17

**Authors:** Na-Jin Gu, Ming-Zhe Wu, Ling He, Xu-Bo Wang, Shiyu Wang, Xue-Shan Qiu, En-Hua Wang, Guang-Ping Wu

**Affiliations:** 1 Department of Pathology, The First Affiliated Hospital and College of Basic Medical Sciences, China Medical University, Shenyang 110001, China; 2 Department of Gynecology, The First Hospital of China Medical University, Shenyang 110001, China; 3 Geisinger Commonwealth School of Medicine; Scranton, PA18510, USA

**Keywords:** Human papillomavirus (HPV), Ras-related associated with diabetes (RRAD), NF-κB, Hypoxia- inducible factor 1α (HIF-1α), glucose transporter 1 (GLUT1)

## Abstract

Chronic infection of HPV16 E6/E7 is frequently associated with lung cancers, especially in non-smokers and in Asians. In our previous studies, we found that HPV16 E6/E7 up-regulated HIF-1α at protein level and further up-regulated GLUT1 at both protein and mRNA levels in well-established lung cancer cell lines. In one of our further mechanism study, the results demonstrated that HPV16 E6/E7 up-regulated the expression of GLUT1 through HPV-LKB1-HIF-1α-GLUT1 axis. However, there are multiple pathways involved in HPV16 E6/E7 regulation of HIF-1α expression. In current study, using double directional genetic manipulation in well-established lung cancer cell lines, we showed that both E6 and E7 down-regulated the expression of RRAD at both protein and mRNA levels. Like LKB1, RRAD is one of the cancer suppressor genes. The loss of RRAD further activated NF-κB by promoted cytoplasmic p65 translocated to nucleus, and up-regulated the expression level of the p-p65 in nucleus. Furthermore, p-p65 up regulated HIF-1α and GLUT1 at both protein and mRNA levels. Thus, we proposed HPV16 E6/E7 up-regulated the expression of GLUT1 through HPV-RRAD-p65- HIF-1α- GLUT1 axis. In conclusion, we demonstrated for the first time that E6 and E7 promoted the expression of HIF-1α and GLUT1 by relieving the inhibitory effect of RRAD which resulted in the activation of NF-κB by promoting cytoplasmic p65 translocated to nucleus, and up-regulated the expression of the p-p65 in nucleus in lung cancer cells. Our findings provided new evidence to support the critical role of RRAD in the pathogenesis of HPV-related lung cancer, and suggested novel therapeutic targets.

## Introduction

In 1979, Syrjänen first hypothesized that HPV infection might play an important role in the occurrence of lung cancer 1. With applying the rapid development of molecular biology techniques to the significant mechanism studies, the investigators showed that HPV16 E6 and E7 proteins were the main oncogenes, and the chronic infection was frequently associated with lung cancers, especially in non-smokers and in Asians 2-5. Recently, we found that HPV16 E6/E7 proteins up-regulated HIF-1α at protein level and further up-regulated GLUT1 at both protein and mRNA levels in four well-established lung cancer cell lines 6. In one of our further mechanism study, the results showed that HPV16 E6/E7 up-regulated the expression of GLUT1 through HPV-LKB1-HIF-1α-GLUT1 axis 6. However, there are multiple pathways involved in HPV16 E6/E7 regulation of HIF-1α expression. Chlon et al showed that E6 proteins inhibited cell apoptosis mainly by degrading p53 gene 7. While Todorovic et al demonstrated that E7 proteins promoted cell proliferation mainly by inhibiting retinoblastoma protein (pRb) 8. It has been reported that RRAD is a direct target gene of p53, which exerts anti-cancer effect in human body 9. Like LKB1, p53, pRB, and RRAD are tumor suppressor genes, therefore, we hypothesized that E6 protein inactivated RRAD by degrading p53 gene. However, the relationship between E7 protein and RRAD has not been reported.

RRAD (Ras-related associated with diabetes) belonged to the Ras-related small GTase family, which was initially identified as a gene associated with type II diabetes and overexpressed in some patients with type II diabetes 10. Wang Y et al showed that the expression level of RRAD protein was decreased in cancer cells with poor prognosis 11. The relationship between the expression level of RRAD and glycolysis was demonstrated by Zhang C et al, their results showed that the ectopic expression of RRAD down-regulated glycolysis, while the knockout of endogenous RRAD up-regulated glycolysis 12. These results strongly indicated that down-regulation of RRAD expression was an important key point for cancer cells to obtain energy supply through glycolysis. Thus, the genes involved in RRAD regulation pathway need to be further investigated.

Recently, two investigative groups showed that RRAD directly bound to p65, a subunit of the NF-κB complex and negatively regulated the activation of NF-κB by inhibiting p65 translocation to the nucleus 12, 13. Among the five members of NF-κB complex: p50, p52, p65, Rel B, and C-Rel, the p50 /p65 heterodimer is the most abundant form of NF-κB complex. The NF-κB complex is inhibited by IκB proteins which trapping NF-κB in the cytoplasm. Phosphorylation and degradation of IκB proteins activated the NF-κB complex which resulted in the translocation of NF-κB complex into the nucleus and bound DNA at kappa-B-binding motif. More importantly, NF-κB phosphorylation played a crucial role in NF-κB directed transactivation and NF-κB phosphorylation controlled transcription was in a gene-specific manner 14.

It had been reported that HIF-1α and GLUT1 were two downstream target genes of NF-κB in esophageal cancer and lung cancer respectively 15, 16. Both genes are involved in glycolysis process by cancer cells and this effect is Warburg effect. The well- known effect indicates that cancer cells consume more glucose for energy supply even under aerobic conditions 17.

In the current study, our results demonstrated that HPV16 E6/E7 up-regulated the expression of GLUT1 through HPV-RRAD-HIF-1α-GLUT1 axis. HPV16 E6/E7 inhibited the expression of RRAD and the loss of RRAD promoted the nuclear translocation of p65 and up-regulated the expression level of p-p65 in nucleus; p-p65 further up regulated HIF-1α and GLUT1 at both protein and mRNA levels.

## Materials and Methods

### Cell culture and plasmids

NCI-H460 and A549 cells obtained from ATCC (Manassas, VA, USA), were cultured in HyClone RPMI 1640 medium. LK2 cells, purchased from the cell bank of Chinese Academy of Sciences (Shanghai, China), were grown in HyClone (Logan, UT, USA) Dulbecco's modified Eagle's medium (DMEM). The culture mediums contained 10% fetal bovine serum at 37°C in a 5% CO₂. The cells were cultured in 6-well plates for 24 hours and ready for the transfection or interference.

Based on our previous study results, NCI-H460 cells were E6 and E7 low-expression cell lines, while A549 and LK2 cells were E6 and E7 high-expression cell lines. The plasmid of pEGFP-N1-HPV16 E6, pEGFP-N1-HPV16 E7, and pEGFP-N1, were kindly provided by Prof Xudong Tang, Institute of Biochemistry and Molecular Biology, Guangdong Medical College, China. HPV16 E6 SiRNA (sc-156008), p65 SiRNA(sc-29410) and control disordered SiRNA (sc-37007) were purchased from Santa Cruz Biotechnology (Santa Cruz, CA, USA). HPV16 E7 SiRNA (Si-h-E7-002), RRAD SiRNA (stB0004153C) were purchased from RIBOBIO (Guangzhou, China). Disordered siRNA was used as a nonspecific siRNA control.

### Transfection and interference

Plasmid with target was transiently transfected into the cells according the instruction provided by Lipofectamine 3000 Transfection Kit (Invitrogen, Carlsbad, CA, USA). Transfection with empty vector and mock transfection served as the controls. The transfection efficiency was evaluated by visualizing the cells expressing Green Fluorescent Protein (GFP) directly under fluorescence microscopy. The protein analysis was assessed 48 hours after transfection by western blotting and immunofluorescent staining. The mRNA analysis was assessed 24 hours after transfection by quantitative real-time reverse transcriptase-polymerase chain reaction (qRT-PCR).

### Western-blot assays

Total protein from cells were extracted in lysis buffer and quantified using the Bradford method. Sixty micrograms of protein were separated by SDS-PAGE, the protein was transferred to polyvinylidene fluoride membrane (Millipore, Billerica, MA, USA). After the membrane was blocked with 5% skimmed milk, the following primary antibodies were applied and incubated separately overnight at 4°C: HPV16 E6 (1:200, Bioss Biotechnology Co., Ltd, Beijing, China), HPV16 E7 (1:200, Bioss Biotechnology Co., Ltd, Beijing, China), RRAD (1:1000; Abcam, Boston, MA, USA), p65 (total protein, 1:1000; Cell Signaling, USA), p-p65 (serine 536 Phosphorylated protein, 1:500; Wanleibio, China), HIF-1α (1:1000; Cell Signaling, USA), GLUT1 (1:500; Wanleibio, China) and GAPDH (1:1000, Cell Signaling Technology, Danvers, MA, USA). The membranes were further incubated with peroxidase-coupled anti-mouse or anti-rabbit IgG (Cell Signaling Technology, Beverly, MA) at 37˚C for 2 hours. The bound proteins were visualized by using ECL (Pierce, Rockford, IL) and the densities were measured by using Bio Imaging System.

### Quantitative real-time PCR assays

Total RNA was extracted from cells using TRIzol Reagent (Life Technologies, Carlsbad, CA, USA) and RNase RNA isolation kit (QIAGEN, Hilden, Germany). First-strand cDNA was synthesized from 500 ng total RNA using high-capacity cDNA RT Kit (Applied Biosystems, Carlsbad, CA, USA). The qRT-PCR was performed using SYBER Green Master Mix on 7900HT Fast Real-Time PCR system (Applied Biosystems) as follows: 95°C for 2min, 40 cycles of 95°C for 15s, 60°C for 60s. A dissociation procedure was performed to generate a melting curve for confirmation of amplification specificity. GAPDH was used as the reference gene. The relative levels of gene expression were represented as ΔCt= Ct gene - Ct reference, and the fold change of gene expression was calculated by the 2^-ΔΔCt^ Method. Experiments were repeated in triplicate. The detailed information of the primers is given in Table 1. The amplified products of E6, E7, RRAD, p65, HIF-1α, GLUT1, and GAPDH were confirmed by DNA gel with correct sizes. The products were extracted, purified from the gel, and sent for DNA sequencing. The sequencing results were 100% correct.

### Immunofluorescence

Cells with 90% confluent were seeded into a 6 well culture plate. After gene transfection or siRNA interference was performed, the cells were cultured for 24 hours. Then 500 ul/well of the cell suspension with a concentration of 5x10^4^ cell/ml was seeded into a 24 well culture plate. Each bottom of the 24 well was pre-inserted with a 15 mm round cover glass slide (Wuxi NEST Biotechnology Co. Wuxi, China) and the cells were cultured for another 24 hours to form a confluent monolayer. The cells were then washed with ice-cold PBS and fixed with methanol. After the permeability treatment with PBS containing 0.2% Triton X-100, thecoverslipswere incubated with anti- p65 antibody (1:50; Cell Signaling, USA) at 4˚C overnight. After incubated with CoraLite488- conjugated Affinipure Goat Anti-Rabbit IgG (H+L) secondary antibody (SA00013-2, PTG, Beijing, China) for 1 h, the coverslips were mounted in Prolong Gold antifade reagent with DAPI (Beyotime, Inc., Shanghai, China), and cover slipped. The cover slips were examined with optical microscope.

### Analysis of p65 nuclear translocation

H460 cells were interference with SiRRAD. The nuclei isolation was performed by using Nuclear and Cytoplasmic Protein Extraction Kit (P0028, Beyotime, China) according to the manufacturer's instructions. The extracted nuclear protein levels of p65 and p-p65 were measured by western blot assays using anti-p65 (Signaling Technology) and p-p65 (serine 536) antibodies (1:500; Wanleibio, China). The level of nuclear histone protein was used as an internal standard and was measured by using anti-Histone antibody (Sangon Biotech). After knockdown of endogenous RRAD, the nuclear translocation of p65 was measured by immunofluorescence.

### Statistical analysis

All statistical analyses were performed using SPSS for Windows 13.0 (SPSS Inc, Chicago, IL, USA). All data presented three or more experiments with similar results.

Statistical significance was determined by Student's t-test, and a p value <0.05 was considered significant.

## Results

### The screening of lung cancer cell lines

Based on our previous results, H460 cell line was low E6 and E7 expression cell line, and A549 and LK2 cells were high E6 and E7 expression cell lines 6. The expression of RRAD and p65 in four lung cancer cell lines, H1299, A549, LK2, and H460 were screened. As shown in S1Fig, high expression of RRAD was found in H460, whereas high expression of p65 was observed in A549 and LK2 cell lines. Further assays were designed and performed based on these results.

### E6 and E7 downregulated the expression of RRAD and upregulated the expression of p65, p-p65, HIF-1α and GLUT1

**Figure 1:**
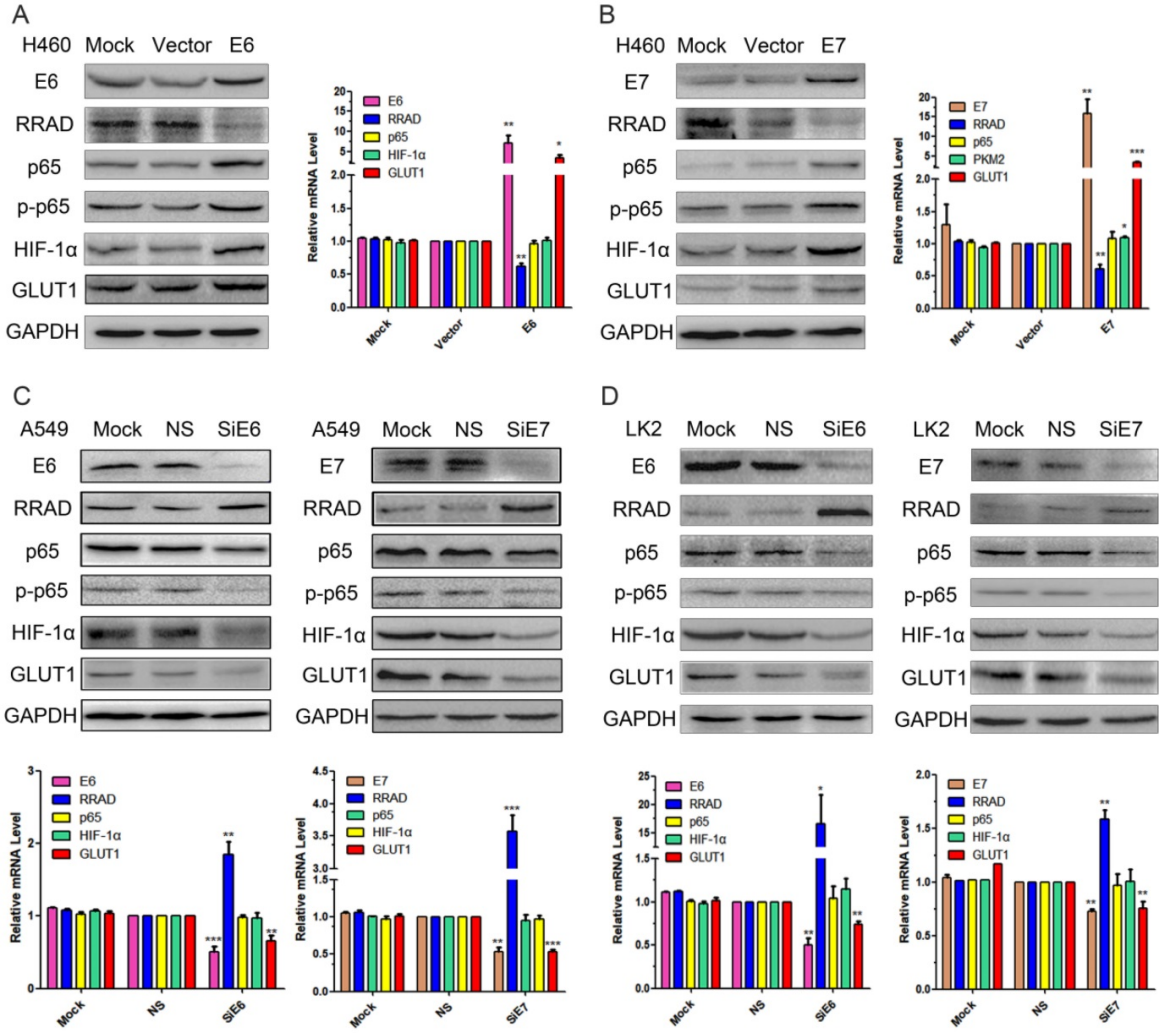
** Effects of E6 and E7 on the regulation of p65, p-p65, HIF-1α and GLUT1 expression in lung cancer cell lines. (A)** The pEGFP-N1-E6 vectors was transfected into H460 cell line, and the E6 empty vectors and mock transfections served as controls. Expression of E6, RRAD, p65, p-p65, HIF-1α, and GLUT1 were demonstrated by western blotting in H460 cells. **(B)** The pEGFP-N1- E7 vectors was transfected into H460 cell line, and the E7 empty vectors and mock transfections served as controls. Expression of E7, RRAD, p65, HIF-1α, and GLUT1 were demonstrated by RT-qPCR in H460 cells. (*p<0.05; **p<0.0l). **(C)** The E6 or E7-specific siRNA were transfected into the A549 cells. E6 or E7-nonspecific siRNA and mock specific siRNA were used to serve as the controls. Expression of E6, E7, RRAD, p65, p-p65, HIF-1α, and GLUT1 were demonstrated by western blotting and RT-qPCR in A549 cells. (*p<0.05; **p<0.0l). **(D)** The E6 or E7-specific siRNA were transfected into the LK2 cells. E6 or E7-nonspecific siRNA and mock specific siRNA were used to serve as the controls. Expression of E6, E7, RRAD, p65, HIF-1α, and GLUT1 were demonstrated by western blotting and RT-qPCR in LK2 cells. (*p<0.05; **p<0.0l).

The pEGFP-N1-E6 or E7 vectors were transiently transfected into the low expression H460 cell line, and the E6 or E7 empty vectors and mock transfections served as controls. The results showed that the overexpression of E6 or E7 significantly downregulated the expression of RRAD at both protein and at mRNA levels, but upregulated the expression of GLUT1 at both levels. The overexpression of E6 or E7 also upregulated the expression of p65, p-p65 and HIF-1α but at protein level only. The results were presented in Fig.1 A and B.

### Knocked down E6 and E7 upregulated the expression of RRAD and downregulated the expression of p65, p-p65, HIF-1α and GLUT1

To further verify the regulatory roles of both E6 and E7 on RRAD, p65, p-p65, HIF-1α, and GLUT1, we applied E6 or E7-specific siRNA to knockdown the expression of E6 or E7 in the A549 and LK2 cell lines. E6 or E7-nonspecific siRNA and mock specific siRNA were used to serve as the controls. The results indicated that the inhibition of both E6 and E7 upregulated the expression of RRAD at both protein and at mRNA levels, but downregulated the expression of GLUT1 at both levels. The inhibition of E6 or E7 also downregulated the expression of p65, p-p65 and HIF-1α but at protein level only. The results were presented in Fig.1 C and D.

### Knocked down RRAD up-regulation the expression of p65, p-p65, HIF-1α and GLUT1

**Figure 2:**
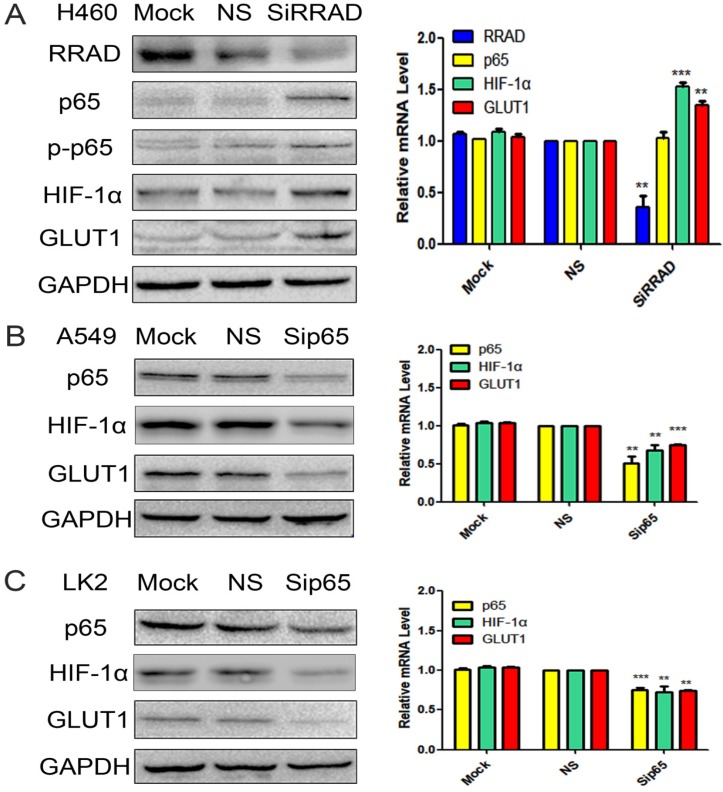
** A) Knocked down RRAD up-regulation the expression of p65, p-p65, HIF-1α and GLUT1.** RRAD-specific siRNA was performed in H460 cells. RRAD-nonspecific siRNA and mock specific siRNA were used to serve as the controls. The relative levels of RRAD, p65, p-p65, HIF-1α, GLUT1 and GAPDH were detected by western blotting and RT-qPCR in H460 cells. **B and C) Knocked p65 downregulated the expression of HIF-1α and GLUT1.** p65-specific siRNA was performed in the A549 and LK2 cell lines. p65-non specific siRNAand mock specific siRNA were used to serve as the controls. The relative levels of p65, HIF-1α, GLUT1 and GAPDH were detected by western blotting and RT-qPCR in A549 and LK2 cells.

High RRAD expression cell line H460 was selected and RRAD-specific siRNA to knockdown the expression of RRAD was performed. RRAD- nonspecific siRNA and mock specific siRNA were used to serve as the controls. The results showed that the inhibition of RRAD significantly upregulated the expression of p65 and p-p65 at the protein level only; and upregulated the expression of HIF-1α and GLUT1 at protein and mRNA levels. RRAD-nonspecific siRNA and mock specific siRNA showed minimal or no change. The results were presented in Fig.2A.

### Knocked p65 downregulated the expression of HIF-1α and GLUT1

p65-specific siRNA to knockdown the expression of p65 in the A549 and LK2 cell lines was performed. p65-nonspecific siRNA and mock specific siRNA were used to serve as the controls. The results indicated that the inhibition of p65 downregulated the expression of HIF-1α and GLUT1 at both protein and mRNA levels. p65-nonspecific siRNA and mock specific siRNA showed minimal or no change. The results were presented in Fig.2B and C.

### Knocked RRAD promoted p65 nuclear translocation

**Figure 3:**
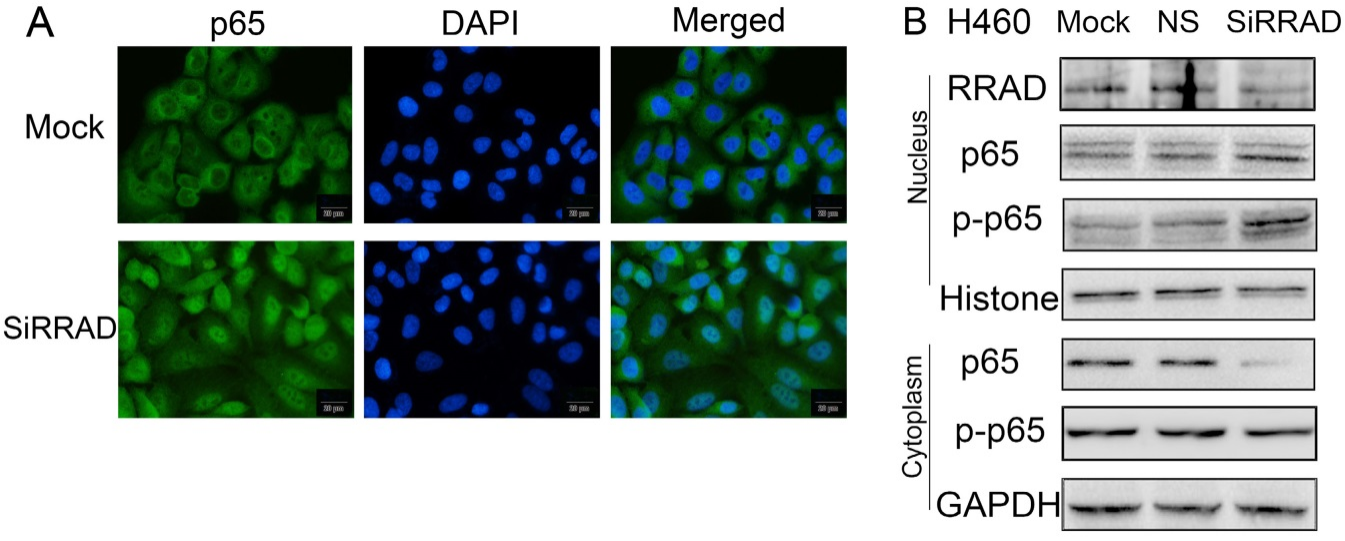
** RRAD knockdown promoted the translocation of p65 to the nuclear. (A)** RRAD knockdown promoted the translocation of p65 to the nuclear in H460 cells. Cells were transfected with RRAD-specific siRNA or mock specific siRNA. Immunofluorescence showed that the inhibition of RRAD promoted p65 nuclear translocation significantly. Scale bar, 20μm. Mock: mock transfection; ns: no significance.** (B)** Knocked RRAD up-regulated the expression levels of p65 and p-p65 in nuclear, but down-regulated the expression level of cytoplasmic p65. RRAD-specific siRNA was performed in H460 cells. RRAD-nonspecific siRNA and mock specific siRNA were used to serve as the controls. Nuclear plasma separation technology was used to separate the proteins in the nucleus from in the cytoplasm. The relative levels of RRAD, p65, p-p65, GAPDH, and Histone were measured by western blotting.

To further verify the regulatory role of RRAD on p65, we knocked RRAD in H460 cells. Our results showed that the inhibition of RRAD promoted p65 nuclear translocation significantly. The assays were performed by immunofluorescence techniques and the results were presented in Fig.3A.

### Knocked RRAD up-regulated the expression levels of p65 and p-p65 in nuclear, but down-regulated the expression level of cytoplasmic p65

We knocked RRAD in H460 cells and we separated the proteins in the nucleus from in the cytoplasm by using nuclear plasma separation technology. We found that the expression levels of p65 and p-p65 in the nuclear were up-regulated, but the expression level of cytoplasmic p65 was down-regulated. The expression level of cytoplasmic p-p65 was almost no change. The results were presented in Fig.3B.

## Discussion

In our previous work, we found that the overexpression of HPV16 E6/E7 up-regulated the expression of HIF-1α which further up-regulated GLUT1 expression in lung cancer cells 6. However, the multiple HPV16 E6 or E7 regulatory pathways on the expression of HIF-1α are unclear. In this study, we showed that the overexpression of E6 or E7 in lung cancer cell lines down-regulated the expression of RRAD at both the protein and mRNA levels. To our knowledge this is the first study found that overexpression of E7 protein down-regulated RRAD. The regulatory mechanism between E7 protein and RRAD is not clear and needs further investigation.

Recently, two investigative groups found that RRAD directly bound to the subunit p65 in the NF-κB complex. The results indicated RRAD negatively regulated the activation of NF-κB by inhibiting p65 entry into the nucleus 12, 13. Another group reported that inhibiting the phosphorylation of p65 subunit in the NF-κB complex negatively regulated the activation of NF-κB 18. Frank et al in the review paper mentioned that NF-κB phosphorylation played a crucial role in NF-κB directed transactivation and NF-κB phosphorylation controlled transcription was in a gene-specific manner 14.

Our results from immunofluorescence and nuclear-cytoplasmic protein separation western blot analysis showed that the inhibition effects of E6 or E7 on RRAD significantly promoted the nuclear translocation of p65 in H460 cells, increased the expression levels of p65 and p-p65 (serine 536) in the nucleus, decreased the expression level of cytoplasmic p65, and the expression of p-p65 in the cytoplasm was almost no change. These results indicated that the activation of NF-κB were triggered by two factors, the nuclear translocation of p65 and increased the expression level of p-p65. It was not clear which happened first, the translocation or the phosphorylation. The detailed steps of the processing need to be further investigated.

Knockdown the expression of p65 in the A549 and LK2 cell lines, we found that the inhibition of p65 down regulated the expression of HIF-1α and GLUT1 at both protein and mRNA levels. The mRNA level regulatory effects by p65 were not consistent with the regulatory effects of interference with E6 and E7 which at protein level only. Our previous studies and Li et al showed the overexpression of E6 and E7 only up-regulated the expression of HIF-1α at protein level, but not at mRNA level 6, 20.

We believed that the mechanism of the regulation might be through translational or post-translational pathways. Our p65 regulatory effects on HIF-1α was consistent with Prangsaengtong et al, they reported that Shikonin significantly down-regulated the expression of HIF-1α at the protein and the mRNA levels after inhibiting p65 19.

In conclusion, we demonstrated for the first time that E6 and E7 promoted the expression of HIF-1α and GLUT1 by relieving the inhibitory effect of RRAD which resulted in the activation of NF-κB by promoted cytoplasmic p65 translocated to nucleus, and up-regulated the expression of the p-p65 in nucleus in lung cancer cells. p-p65 further upregulated the expression of both HIF-1α and GLUT1 at the protein and the mRNA levels. Therefore, E6 and E7 regulate the expression of both HIF-1α and GLUT1 through HPV-RRAD-p65-HIF-1α- GLUT1 axis. Our findings provided new evidence to support the critical role of RRAD in the pathogenesis of HPV-related lung cancer, and suggested novel therapeutic targets.

## Figures and Tables

**Table 1:** Sequencesand features of primers used for qRT-PCR

Gene	Forward/ Reverse	Sequence	Size (bp)	mRNA
E6	270	GTATGGAACAACATTAGAACAGCAA	79	KX545363
	349	GTGGCTTTTGACAGTTAATACACC		
E7	482	GCATGGAGATACACCTACATTG	273	KX545363
	754	TGGTTTCTGAGAACAGATGG		
RRAD	785	TGAGGTCTCGGTGGATGAGG	74	NM_001128850.1
	945	GGTGCTCAGGGATGACGTAAA		
P65	926	GCAGGCTCCTGTGCGTGTCT	286	NM_021975.3
	945	GGTGCTCAGGGATGACGTAAA		
HIF-1α	1811	AGACAAAGTTCACCTGAGCC	174	NM_001530.4
	1984	GGGAGCTAACATCTCCAAGTCT		
GLUT1	1071	CTGGCATCAACGCTGTCTTC	167	NM_006516.3
	1237	GCCTATGAGGTGCAGGGTC		
GAPDH	50 120	TTCTTTTGCGTCGCCAGCCGAG CCAGGCGCCCAATACGACCAAA	71	XM_019023188.1


mRNA: messenger RNA; qRT-PCR: quantitative real-time reverse transcriptase-polymerase chain reaction
